# Literary Journals

**Published:** 1868-07

**Authors:** 


Literary Journals.—*The Atlantic Monthly*, never failing in its regular appearance and never failing in interest and instruction, is to be commended to all lovers of intellectual pleasure and progress. The August number contains for its first article "A Remarkable Case of Physical Phenomena," to which we call the attention of our readers.

*The Nation*, as a secular and political paper stands first in the world, and we are always glad to exchange a little medicine for so much common sense in politics and variety in everything which pertains to the common and public affairs of the world.

